# A Case Study of Malrotated Kidneys with Asymmetric Multiple Renal Arteries, Variant Venous Drainage, and Unilateral Ureteral Duplication

**DOI:** 10.1155/2019/1893137

**Published:** 2019-03-18

**Authors:** Martine Dunnwald, Marc A. Pizzimenti

**Affiliations:** Department of Anatomy and Cell Biology, Roy J. and Lucille A. Carver College of Medicine, University of Iowa, Iowa City, Iowa, USA

## Abstract

Variations in the arterial, venous, and ureteral patterning of the right (r) and left (l) kidneys are common; however, concomitant involvement with all three systems is rare. Specimens that demonstrate anatomic variation across multiple systems provide an opportunity to illustrate links between anatomic concepts, embryologic development, clinical practice, and education. During anatomic study of the abdominal cavity, a total of five major arteries (3l and 2r) emerged from the aortic and common iliac axes in a cadaveric donor. Through continued study, multiple contributing veins, of different caliber, coalesced into four major renal veins (2l and 2r) that returned blood from the kidneys to the inferior vena cava (IVC) at different locations. In addition, unilateral duplication of the kidney with concomitant ureters was evident on the right side. Both ureters continued inferiorly and independently entered the bladder, each with an observable orifice adjacent to the bladder trigone. Most evident in the specimen was the anteriorly directed hilum for both kidneys. Reported measures for each of the observed anatomic variations suggest that the current specimen has an estimated incidence of less than 0.3%. This comparatively rare specimen provides an example of important anatomic concepts that are relevant to educational and clinical practices.

## 1. Introduction

Although variations in the arterial, venous, and ureteral patterning of the kidneys are common, concomitant involvement of variant anatomy in all of these systems appears to be much rarer. Arterial variants that diverge from the generalized pattern of paired renal arteries emerging laterally from the aorta, inferior to the level of the superior mesenteric artery, have been observed in approximately 30% of individuals (e.g., [1, 2]). In the venous system, variations in the drainage pattern that diverge from the classically described paired vessels flowing into the inferior vena cava (IVC) have been well documented and can have an incidence rate similar to that of the arterial system (e.g., [3]). However, variation in the ureteral pattern is not as common as that found in the vascular system. Reports indicate that genetic factors contribute to ureteric variations [4, 5] which occur with an approximate incidence of 1% [6, 7].

The case study presented here demonstrates concomitant variation in the arterial, venous, and ureteric systems of the kidney. Collectively, this case provides examples of important anatomic considerations that relate the foundational anatomical science with educational practice. These include the generalized concept of anatomic variation, the process of renal morphogenesis with variant presentations, and considerations for clinical procedures with involved variant anatomy.

## 2. Case Report

During anatomic study of the abdominal cavity, additional renal branches from the aortic axis were revealed in a 97-year-old male Caucasian cadaveric donor. Continued dissection outlined notable anatomic variations in the arterial, venous, and ureteric patterns. No urologic or cardiovascular issues were reported by the donor or family at the time of enrolment in the Deeded Body Program. For this type of study, Institutional Review Board approval is not required for research conducted with cadaveric specimens.

**Figure 1:**
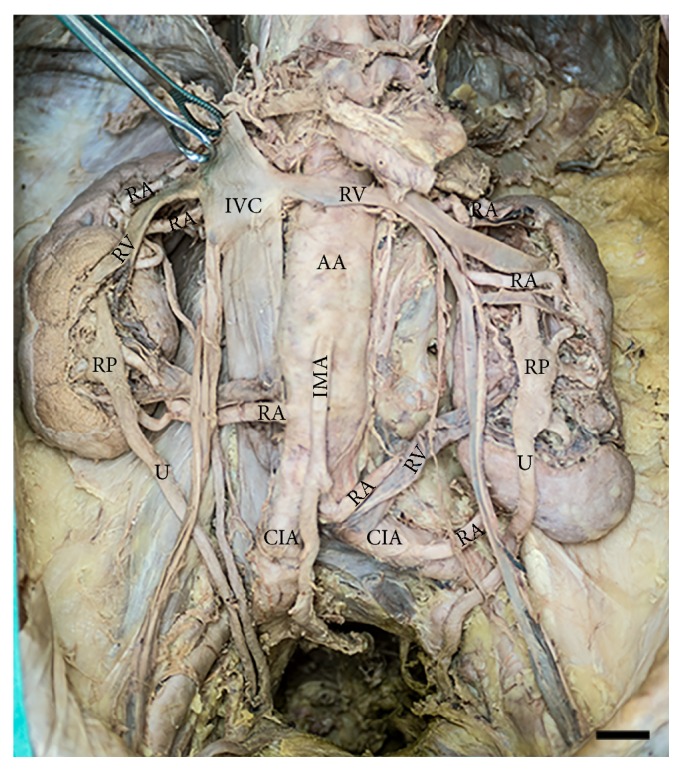
*Renal system in situ*. Macroscopic view of the renal system within the abdominal cavity of the donor. AA = abdominal aorta, CIA = common iliac artery, IMA = inferior mesenteric artery, IVC = inferior vena cava, RA = renal artery, RP = renal pelvis, RV = renal vein, U = ureter. Image scale bar = 2 cm.

Both kidneys were retroperitoneal and similar in size with measurements of 12.3 cm (l) and 12.7 cm (r) in the craniocaudal direction. Despite the fact that the lengths were similar, there were marked differences in the relative positioning of the superior and inferior poles. The superior pole of the right kidney was situated more superiorly. The inferior pole of the left kidney was positioned near the superior border of the left common iliac artery. Although each kidney occupied an extended volume, neither kidney had a pelvic component. Hilar structures entered/exited the organs anteriorly, not with the typical medially projected hilum (Figure 1).

### 2.1. Arterial Pattern

**Figure 2:**
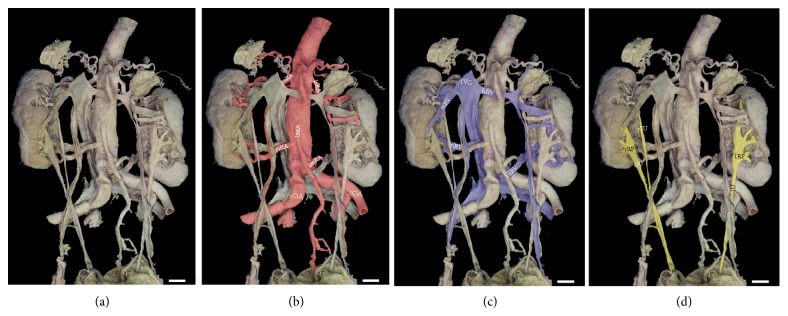
*Ex situ renal system exhibits anatomical variations from normal pattern*. (a) Uncolorized renal system* ex situ.* (b) Arterial network pseudocolorized in red. rsRA = right superior renal artery, lsRA = left superior renal artery, IMA = inferior mesenteric artery, riRA = right inferior renal artery, lmRA = left middle renal artery, liRA = left inferior renal artery, rCiRA = right common iliac artery, lCiA = left common iliac artery. (c) Venous network pseudocolorized in blue. IVC = inferior vena cava, rsRV = right superior renal vein, lsRV = left superior renal vein, riRV = right inferior renal vein, liRV = left inferior renal vein. (d) Ureters pseudocolorized in yellow with rRP = right renal pelvis, lRP = left renal pelvis, rU = right ureters, lU= left ureter. Pseudocolorization created using Adobe Photoshop. Image scale bar = 2 cm.

**Figure 3:**
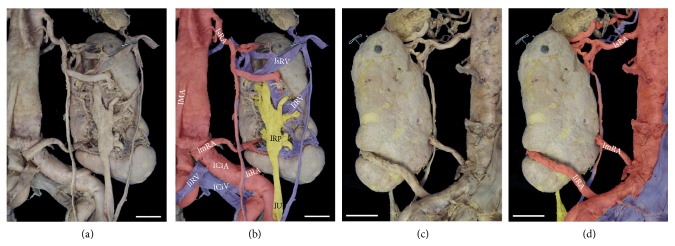
*Ex situ left kidney exhibits anatomical variations*. Anterior (a, b) and posterior (c, d) views of the left kidney. Arterial, venous and ureteric systems (b, d) are pseudocolorized in red (arterial), blue (venous) and yellow (ureteric). Renal veins (lsRV, liRV) are reflected to better illustrate the arterial pattern. Image scale bar = 2 cm.

**Figure 4:**
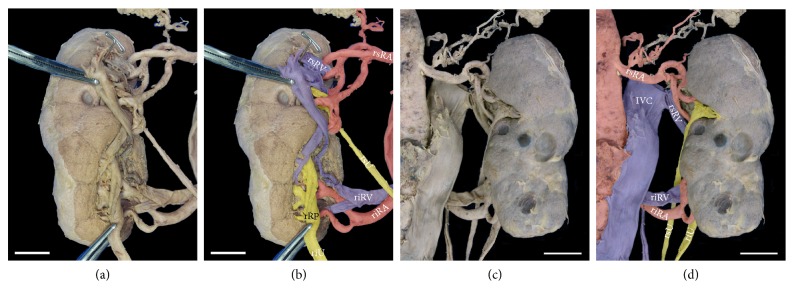
*Ex situ right kidney exhibits anatomical variations*. Anterior (a, b) and posterior (c, d) views of the right kidney. Arterial, venous, and ureteric systems (b, d) are pseudocolorized in red (arterial), blue (venous), and yellow (ureteric).

A total of five major arteries (3l and 2r) emerged from the aortic and common iliac axes (Figures 2(a) and 2(b)). The superior left renal artery originated from the abdominal aorta and supplied the superior pole. This artery (5.2 mm diameter) branched into two vessels of similar caliber to supply the upper third of the organ. Supply to the left gonad originated from the more inferior of these two branches (Figures 2(b) and 3(b)). The middle left renal artery (6.1 mm diameter) originated from the iliac junction, just anterior to the median sacral artery (Figures 3(a) and 3(b)). The inferior left renal artery (5.4 mm diameter) originated from the common iliac artery, and coursed posteriorly to the kidney before entering the hilum anteriorly (from the lateral aspect of the organ), to supply the inferior pole (Figures 3(b), 3(c), and 3(d)). On the contralateral side, the vasculature to the right kidney consisted of only two renal arteries, both originating from the abdominal aorta. The superior renal artery (6.2 mm diameter) branched laterally from the aorta (at the same level as the left superior renal) and supplied the superior pole. As with the contralateral side, this artery also branched into two vessels of similar caliber, but supplied the superior half of the organ (Figures 4(a) and 4(b)). In contrast to the contralateral side, the right gonadal artery branched directly from the aorta at its generally observed position, just inferior to the (superior) renal artery. Arterial supply to the inferior pole (6.5 mm diameter) originated on the lateral aorta, at a level inferior to the inferior mesenteric artery, and branched into two arteries (Figures 4(b), 4(c), and 4(d)).

### 2.2. Venous Pattern

Multiple contributing veins, of different caliber, coalesced into four major renal veins (2l and 2r) that returned blood from the kidneys to the IVC (Figure 2(c)). The relative positioning of the superior renal veins followed the conventional pattern, entering the IVC through single vessels at a level just inferior to the superior mesenteric artery as it emerged from the aorta. On the right side, three major veins merged into a short segment (3 mm in length) to drain the superior 2/3 of the organ. The inferior 1/3 of the organ was drained through two primary contributors that merged into a single vein that ultimately drained into the IVC at the level of the previously described right inferior renal artery. A visible and substantive anastomotic connection was evident between the superior and inferior venous pathways (Figure 3(b)). On the left side, the superior renal vein received contributions from the suprarenal gland and the superior half of the kidney. Venous return from the left gonad ultimately merged into the most inferior branch of the three primary contributors to the superior renal vein (Figures 2(c) and 3(b)). The inferior renal vein drained directly into the anterior aspect of the IVC at the junction of the common iliac veins, posterior to the bifurcation of the aorta. The most inferior of the three primary contributors to the inferior renal vein emerged from the posterior lateral aspect of kidney and spanned the hilum to ultimately converge into the inferior renal vein. The middle contributor emerged from the parenchyma, but was unremarkable. As on the contralateral side, a visible and substantive venous anastomotic connection was evident between the superior and inferior aspects of the kidney (Figure 4(b)).

### 2.3. Ureteral Pattern

Unilateral duplication of the kidney with concomitant ureters was evident for the right side (Figure 2(d)). Aligned with the arterial pattern of this organ, the superior ureter demonstrated a discreet collecting system with well-formed minor and major calyces draining into a defined renal pelvis. The majority of the superior renal pelvis was positioned posterior to the vasculature (Figures 4(a) and 4(b)). At the inferior portion of the kidney, the hilum was more anteriorly directed with the calyces and renal pelvis more evident given their anterior position relative to the vasculature. Both ureters continued inferiorly and independently to enter the bladder (Figure 1). Histological investigation of the ureterovesical junctions was not completed; however a distinct orifice near the bladder trigone was evident for each ureter. On the contralateral side, the single ureter emerged from a widened and anteriorly projected hilum (Figure 3(b)). Major calyces from the peripheral parenchyma were evident and these calyces contributed to an elongated renal pelvis. The remaining course of the left ureter was unremarkable.

## 3. Discussion

Rare case studies present opportunities to not only consider the observed morphological variations but also revisit underlying concepts in embryology and the associated educational implications. Variations in the arterial, venous, and ureteral patterning of the kidneys are common; however, involvement with all three systems is very rare. The current case demonstrates concomitant variation in the arterial, venous, and ureteral systems, which is conservatively estimated to have an incidence of less than 0.3%.

### 3.1. Anatomic Variation

Arterial variants have been observed in approximately 30% of individuals, although population specific incidences have been shown to vary considerably [1, 8]. Studies documenting the major renal branches, rather than the branching pattern into kidney parenchyma, suggest that arterial origins along the entire abdominal aortic axis are possible. Branches at or superior to the level of the superior mesenteric artery [9] and along the lumbar region [10–14] have been well documented. Arterial branching from the iliac axis has also been documented, particularly in ectopic kidneys [15–17], however ectopic specimens without pelvic involvement have also demonstrated branches arising from the iliac region [14, 18]. Bilateral variation in the arterial patterning has been quantified with an incidence of approximately 10% [19] while unilateral arterial supply to the kidney arising from all of these regions has a suggested incidence of approximately 0.4% [20, 21].

Although early modern attempts to quantify patterning through direct patient (and/or cadaveric) observation (e.g., [22–24]) were compelling, more recent studies using imaging (e.g., [18, 19, 21]) have verified and improved our understanding of the incidence for these arterial patterns. They have been classified in Patterns (I-V) and Types (A-E) based on the number of vessels observed and the location from which the vessels arise, respectively. From these studies, the arterial distribution to the right kidney in the current specimen generally fits the description for Pattern II and Type D, where two arteries enter the kidney and the inferior renal segment is supplied directly from the aorta. This arterial pattern/type has an incidence rate of approximately 2%. The left kidney in the current specimen most closely demonstrates Pattern III, with 3-4 distinct arteries, but the Type descriptor falls outside of the general classification schema. Here, the current specimen is most similar to case studies presented through direct observation in cadaveric specimens [25] or visualized through CT-3D reconstruction [18]. Although Cases et al. did not include a reconstructed case in the analysis, the data set would suggest an hypothesized incidence for the current arterial pattern to be at minimum of 0.4%. However, a statistically sound meta-analysis that includes specific branching categories across studies has yet to be completed.

As with the arterial system, variation in renal vein patterning is not uncommon. Studies with large sample sizes (e.g., >100) suggest that variation in venous patterning has an incidence of 23.5-30%, as observed through computed tomography (CT) [3, 26–28]. This reported incidence has also been observed in 105 cases of horseshoe kidney [29]. Given the high incidence of renal vein variation, a recent study used CT to observe and classify these variations within a comparatively large sample size (1452 patients) [28]. In this classification system, the venous drainage of the right kidney in the current specimen could be described as Type 2A (0.3% incidence), with direct drainage into the IVC from two renal veins. The venous drainage of the left kidney could be described as Type IB (1.4% incidence), with drainage into the IVC and common iliac vein. What is unclear from these studies is if the incidence rates are applicable for bilateral variations.

Variation in ureteral patterning is not as common as that found in the vascular system, but reports indicate an approximate incidence of 1% [6, 7], with unilateral duplex kidney reported as high as 1.8% [30]. Incidence of ureteral duplication appears to have a familial pattern of inheritance [4, 5] that, for a duplex kidney, results from more than one ureteric bud outgrowth from the mesonephric duct during fetal development.

As outlined above, aspects of the variations described in the current case have been observed elsewhere. However, few of those cases have provided detailed descriptions and illustrations of concomitant involvement of each system. Collectively, this case appears to be rare given the low incidence rates for each (independent) system: arterial, 0.4%; venous, 0.3-1.4%; and ureteral, 1-1.8%. Based on this review of studies, and using the reported systems-related variation, the incidence rate for the current specimen is conservatively estimated at less than 0.3% of the population.

### 3.2. Educational and Embryological Considerations

Students who participate in dissection courses early in their training, or students rotating through their clinical training, learn the basic premise of anatomic variation—that organs/regions under study often do not appear, at least at first, as outlined in a referenced source. Distinguishing which variations have clinical significance requires both breadth and depth of training, particularly within each clinical specialty [31]. Although exposure to the concept of variation usually occurs early in training and primarily through dissection courses [31], many of the region/organ details are often revisited within clinical clerkships and during residency training [32]. For example, variations in renal arterial patterning may have little clinical significance unless they are coupled with hypertension or require mapping for clinical procedures such as surgical resection (e.g., [33]), transplantation (e.g., [34]), or percutaneous access (e.g., [35]). Given the multisystem (i.e., arterial, venous, and ureteral) involvement, this case clearly demonstrates the anatomical aspect of this concept.

**Figure 5:**
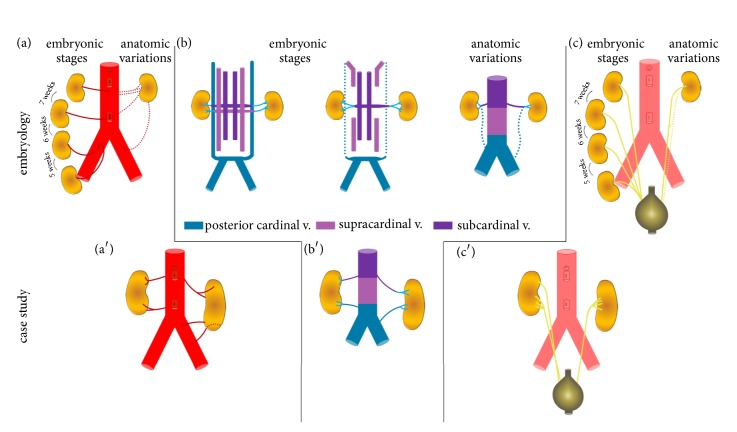
*Simplified schematic representation of renal development*. Around 5 weeks of fetal life, kidneys are formed from the metanephros. They are situated at the level of the pelvis and pelvic branches of the common iliac artery provide initial arterial blood supply. During the following 3 weeks, the kidneys ascend in the abdominal cavity (a) to their adult retroperitoneal location. As they move cranially and the metanephros coalesces, the lower arteries degenerate to, most often, leave a single renal artery as the main arterial supply. Occasionally, the temporary arteries (dotted lines (a)) do not degenerate leading to anatomical variations in the adult (a′). The cardinal systems contribute to the developing venous network of the kidney as it ascends (b). Persistence of some aspects of the developing venous network may lead to anatomical variations in the adult (dotted lines (b), (b′)). More than one ureteric bud may penetrate the metanephric mesoderm during ureteral development (c) that results in duplex kidney (left (c′)).

Collectively, the anatomic variations of the current case also reflect important concepts related to the morphological development of the kidney and its collecting system. During human development, three slightly overlapping renal systems are formed in a cranial to caudal fashion: pronephros, mesonephros and metanephros [36]. The pronephros is a temporary organ that regresses at the end of the fourth week of fetal life while the mesonephros contributes to Bowman's capsule and the Wolffian duct in adult kidneys. The metanephros appears in the fifth week of fetal life and will give rise to the adult kidney. The renal excretory units originate from the metanephric mesoderm and collecting ducts from the ureteric bud that penetrates the mesodermic tissue. While the primordial kidneys are initially located within the pelvis with their hila projected anteriorly to receive their arterial supply from a pelvic branch of the aorta, this orientation changes with development. Following growth and change in body length at the lumbar and sacral regions, the kidneys undergo a relative “ascent” in the abdomen as they shift to a more cranial position [37, 38]. This ascent is accompanied by a transitioning arterial supply originating from aortic branches at sequentially higher levels and medial rotation of the developing kidney, as schematically depicted in Figure 5(a). The lower vessels usually degenerate, but occasionally, some remain, leading to anatomical variations in the adult (Figures 2(b), 3(b), 3(c), 4(b), 4(d), and 5(a′)).

Unlike the arterial system, the venous system for the kidney arises from anastomotic connections between the developing cardinal veins [38]. Briefly, at 5-6 weeks of development the posterior and subcardinal veins exhibit anastomotic connections in a segment-like fashion that drain aspects of the early mesonephros. The subcardinal veins anastomose and coalesce in the midline, contributing to the prerenal portion of the IVC and what is generally considered the developed right renal vein. Supracardinal veins, like the other renal contributors, begin as bilaterally symmetric having segmental connections with the posterior cardinal veins. As development continues, the posterior cardinal veins degenerate, losing the segmental nature of the connections that were established earlier with the subcardinal and supracardinal network. Ultimately, the supracardinal system contributes to the postrenal segment of the IVC. This segment fuses with caudal posterior cardinal segment that, in turn, gives rise to the common iliac veins. Concomitantly, the supracardinal system on the left side loses connections with the developed left renal vein and the hemiazygos system. Even with this simplified overview of a very complex developmental process (Figure 5(b)), the persistent connections between the venous cardinal networks (Figures 2(c), 3(b), 4(b), and 5(b′)) can be observed in the current specimen.

Development of the ureters arises from bud outgrowths of the mesonephric duct. For each kidney, the ureteric bud penetrates the metanephric mesoderm (metanephrogenic blastema) that forms a cap into which the renal pelvis, calyxes, and collecting tubules develop through branching morphogenesis [36]. The stalk of the ureteric bud elongates as the kidney undergoes its relative ascent resulting in the formed ureter by the ninth week of development (Figure 5(c)). The simultaneous morphogenesis of the vascular and ureteric structures influences the medial rotation of the hilum for each developing kidney. Details of the signaling pathways and the genes that regulate this entire process have been effectively summarized in the literature [39, 40] and are beyond the scope of this case study. These processes coupled with genetic factors may give rise to variations in the ureteric pattern (Figures 2(d), 4(b), 4(d), 5(c), and 5(c′)).

## 4. Conclusion

Anatomic variations in the systems of the developing kidney are common; however, concomitant involvement of the arterial, venous, and ureteric systems is rare. The current case study demonstrates a cadaveric specimen with anatomic variations having an estimated incidence of less than 0.3%. Collectively, this case illustrates examples of important anatomic concepts that are relevant for educational and clinical practice.
